# Editorial: Inter- and Intra-subject Variability in Brain Imaging and Decoding

**DOI:** 10.3389/fncom.2021.791129

**Published:** 2021-11-29

**Authors:** Chun-Shu Wei, Corey J. Keller, Junhua Li, Yuan-Pin Lin, Masaki Nakanishi, Johanna Wagner, Wei Wu, Yu Zhang, Tzyy-Ping Jung

**Affiliations:** ^1^Department of Computer Science, National Yang Ming Chiao Tung University, Hsinchu, Taiwan; ^2^Institute of Education, National Yang Ming Chiao Tung University, Hsinchu, Taiwan; ^3^Institute of Electrical and Control Engineering, National Yang Ming Chiao Tung University, Hsinchu, Taiwan; ^4^Department of Psychiatry and Behavioral Sciences, Stanford University, Stanford, CA, United States; ^5^School of Computer Science and Electronic Engineering, University of Essex, Colchester, United Kingdom; ^6^Institute of Medical Science and Technology, National Sun Yat-sen University, Kaohsiung, Taiwan; ^7^Department of Electrical Engineering, National Sun Yat-sen University, Kaohsiung, Taiwan; ^8^Institute for Neural Computation, University of California, San Diego, San Diego, CA, United States; ^9^Department of Bioengineering, Lehigh University, Bethlehem, PA, United States

**Keywords:** human variability, neuroimaging, brain decoding, brain-computer interface, EEG, MEG, fMRI, fNIRS

Pervasive and elusive human variability, both across and within individuals, poses a major challenge in interpreting and decoding human brain activity. Individual differences in brain anatomy and function contribute to inter-subject variability. A variety of factors may contribute to intra-subject variability, including neural processing, brain activity non-stationarity, neurophysiological mechanisms, and certain unknown factors.

Studies have recently focused on embracing variability rather than disregarding it. By focusing on variability, they have improved insights into individual differences and cross session variations, enabling precise mapping and decoding of functional brain areas based on individual variability and similarity. For instance, transfer learning techniques have enhanced brain decoding performance by dealing with variations in data collected from different subjects over a wide range of sessions and days. The applicability of a neurophysiological biometric is determined by its manifest inter-subject variability and minimal intra-subject variability. As a result, questions arise about how to observe, analyze, and model inter- and intra-subject variability, what researchers might gain or lose from this variability, and how to cope with the variability in brain imaging and decoding.

This Research Topic emphasizes the need to account for both inter- and intra-subject variability in brain imaging and decoding. The present collection contains an expanded overview of related fields and can shed light on future endeavors in those fields. We highlight three domains in this editorial that emerge from the sixteen contributions of this topic:

(1) Characterizing inter- and intra-subject variability in neural observations
(2) Analyzing and assessing the variability of neural data
(3) Methods for eliminating inter- and intra-subject variability in brain imaging and decoding

Our editorial cannot fully encapsulate all the details and depth of this Research Topic. As such, we encourage you to peruse these articles to gain a fuller understanding of the research field of brain imaging and decoding.

*1. Characterizing inter- and intra-subject variability in neural observations*

A collection of contributions exhibits a wide range of characteristics of inter- and intra-subject variability in various types of neural observations including sensorimotor electroencephalographic (EEG) pattern, cerebral metabolism, clinical neuromarkers, brain structure, etc. Ma et al. assessed the cerebral structural changes associated with the effect of chronic pain on empathy, and identified multiple structural brain abnormal pathways connected to anterior insula in a population of patients suffering from chronic lower back pain. Shen and Lin showed that emotional responses exhibited salient intra- and inter-individual differences and considerably modulated the spatio-spectral EEG oscillations. Such EEG variability may lead to a great challenge for the development of a generalized emotion-classification model for real-life applications.

The presence of inter- and intra-subject variability has a significant impact on the findings in neurobiological studies. Sundar et al. investigated whether or not functional connectivity can be integrated to reduce the variability of absolute values of the cerebral metabolic rate of glucose (CMRGlc) and showed that functional connectivity among six major brain networks was not suited for standardization of CMRGlc values. Cai et al. measured the interaction effect on frontal-striatum-thalamus by rs11146020 and rs3813296 from GRIN1 and GRIA2 genes in first-episode negative schizophrenia patients. Their results suggested a modulation on the glutamic frontal-striatum-thalamus pathway by rs11146020 and rs3813296 gene polymorphism. According to the findings, patients with different genotypes have different neuroimaging characteristics on causality connections and structural characteristics in the frontal-striatum-thalamus pathway, implying the importance of personalized clinical interventions.

The performance of using brain-computer interfacing (BCI) systems varies greatly across subjects. Saha and Baumert addressed an important issue of varying neurophysiological processes in sensorimotor rhythms over time and across subjects. They found that time-variant and individualized neurophysiological characteristics could have a significant impact on BCI performance. Lee et al. applied a dynamic causal modeling method to study how motor networks measured by EEG during the resting state could predict the performance of motor imagery. They discovered a significant difference in the connectivity strength from the supplementary motor area to the right dorsolateral prefrontal cortex between the low- and high-performance groups. These findings advanced the understanding of the inefficiency of BCI and the prevention of ineffective use of BCI.

*2. Evaluation and assessment of the variability in neural data*

Following the papers that characterize inter- and intra-subject variability in neural observations, another set of papers focuses on evaluating and assessing the variability in their neural data. Goodman et al. assessed the stability of a mildly stress-inducing math calculation task to evaluate its usability in clinical trials aimed at reducing stress responses. They found good stability in most of the functional magnetic resonance imaging (fMRI) measures performed twice, 13 weeks apart. The authors measure and show significant test-retest reliability of neuronal activation and physiological responses associated with acute psychosocial stress using Montreal Imaging Stress Tasks. Wriessnegger et al. evaluated inter-individual differences in event-related desynchronization/synchronization (ERD/S) patterns during sports motor imagery. The correlation distance of ERD/S values in six region-of-interests between pairs of participants was used to assess inter-individual differences. Mikkelsen et al. addressed the variability between EEG recordings collected in lab and at home locations. Thanks to the emergence of wearable EEG devices, EEG data collection could be performed outside the lab. They concluded that while an experimental environment can affect the quality of EEG data, the effect is smaller than the natural inter-individual variances. The data is thus valid to use from an experimental perspective. The findings would encourage researchers to collect EEG measurements at home.

*3. Methods for obviating inter- and intra-subject variability in brain imaging and decoding*

The last group of contributions sheds light on techniques for obviating inter- and intra-subject variability in analyses of brain imaging and/or decoding brain activities. Yang et al. proposed a neural network with error feedback to improve the stability of neural signal decoding, which is critical for the performance of brain-machine interfaces. The results showed that using an evolutionary network with error feedback could improve decoding stability significantly, compared to either the same network without error feedback. Xu et al. addressed the problem of variability across EEG datasets, which led to a model generalization in EEG classification. To address the aforementioned issue, a pre-alignment strategy, in which covariance matrices were aligned, was proposed to mitigate the variability problem. The alignment used in the study effectively reduced the variability across EEG datasets and improved the performance of cross-dataset classification. The comparison results demonstrated that this strategy could be promising for improving EEG classification accuracy across datasets.

Liang and Liu studied how emotions perceived from whole-person (all facial and body parts included) stimuli could be decoded using motion-sensitive areas. Results revealed that emotions could be successfully decoded based on the activation patterns in dorsal motion-sensitive areas. Furthermore, results from the cross-subject classification analysis showed that motion-sensitive areas supported the classification of individual emotion representation across subjects. Their findings provide new evidence for the involvement of motion-sensitive areas in emotion decoding, and they also suggested that there exists a common emotion code in the motion-sensitive areas across individual subjects. Kanoga et al. presented an automatic artifact reduction technique based on independent low-rank matrix analysis (ILRMA), which was compared to independent component analysis (ICA) and independent vector analysis (IVA) on a public EEG dataset containing various BCI paradigms. The results suggested that ILRMA has the potential to achieve higher discriminability than ICA and IVA for BCIs.

Nonetheless, the presence of variability in brain imaging/decoding gives rise to a new class of techniques that leverage the variability to gain insight into their neural data. Wang et al. proposed a novel method using individual template-based multivariate synchronization index and adaptive threshold strategy for high-speed SSVEP-BCI. Trinh et al. investigated the use of task-induced intra-subject variability of resting-state EEGs for the classification and early detection of individuals with mild cognitive impairment (MCI) and Alzheimer's disease (AD). The results showed that the between-run spectral power similarity/variability could provide better performance than single-run resting-state EEGs. Qiao et al. used machine-learning approaches to extract biomarkers from resting EEG signals and identify the differences between major depression disorder (MDD) and healthy control (HC) groups.

## Author Contributions

C-SW: coordinated the writing process and proposed the structure of the article. All authors contributed equally to the editing process.

## Funding

This work was supported by the Ministry of Science and Technology of Taiwan (MOST 109-2222-E-009-006-MY3, 109-2221-E-110-009-MY3), the Higher Education Sprout Project of the National Yang Ming Chiao Tung University and Ministry of Education of Taiwan, the US Army Research Laboratory (W911NF2120154), the US National Science Foundation (CBET-1935860), the US National Institute of Mental Health (R01MH126639 and R01MH129018), Burroughs Wellcome Fund (Career Award for Medical Scientists), a gift to UC San Diego from The Swartz Foundation (Sag Harbor, NY), the National Natural Science Foundation of China (61806149), and the Guangdong Basic and Applied Basic Research Foundation (2020A1515010991).

## Conflict of Interest

The authors declare that the research was conducted in the absence of any commercial or financial relationships that could be construed as a potential conflict of interest.

## Publisher's Note

All claims expressed in this article are solely those of the authors and do not necessarily represent those of their affiliated organizations, or those of the publisher, the editors and the reviewers. Any product that may be evaluated in this article, or claim that may be made by its manufacturer, is not guaranteed or endorsed by the publisher.

